# Precision Cervical Reconstruction: A State-Dependent Operative Framework for Cervical Insufficiency

**DOI:** 10.3390/diagnostics16203389

**Published:** 2026-10-18

**Authors:** Yong-Jin Park, Moon-Il Park

**Affiliations:** 1 Department of Obstetrics and Gynecology, Dongtan Jeil Women’s Hospital, Hwaseong 18450, Republic of Korea; paladinjin8@gmail.com; 2 Department of Obstetrics and Gynecology, Hanyang University Hospital, Seoul 04763, Republic of Korea

**Keywords:** cervical insufficiency, cervical cerclage, emergency cervical cerclage, precision surgery, cervical biomechanics, operative state, double-level cerclage, cervical elastography, postoperative ultrasound, achieved reconstruction

## Abstract

Cervical insufficiency is conventionally classified by indication and named operative technique, yet neither fully describes the cervix as a dynamically remodeling, mechanically loaded, and structurally heterogeneous organ or specifies the reconstruction delivered. This Perspective proposes precision cervical reconstruction, a conceptual state-informed framework linking five domains: dynamic cervical phenotype, state-based operative assessment, mechanical objective, intended configuration, and achieved reconstruction. Safety and biological eligibility constrain the entire pathway. In the ultrasound-indicated context, morphology and trajectory complement cervical length, whereas elastography and other biomechanical measurements remain research-oriented. In emergency states, bulging extent, membrane reducibility, and cervical rim adequacy separately describe procedural complexity and feasibility. The framework distinguishes clinical observation, plausible mechanical interpretation, and testable hypothesis; it does not replace established indications or constitute a validated treatment-selection algorithm. Suture height, axial separation, sequence, material, tissue engagement, and membrane relationship are treated as reportable variables, and double-level cerclage is considered one conditional configuration rather than a universally superior strategy. Current comparative evidence does not establish consistent benefit from an additional suture or identify a validated state in which vertically distributed support improves outcomes. The immediate contribution is a standardized reasoning and reporting structure that separates indication from state, intended configuration from achieved reconstruction, and mechanistic hypotheses from clinical effectiveness. Prospective studies should establish measurement reliability, procedural fidelity, safety, and associations with maternal and neonatal outcomes before testing state-guided care.

## 1. Introduction: From Cerclage to Precision Cervical Reconstruction

Transvaginal cervical length is strongly associated with spontaneous preterm-birth risk, and cervical cerclage remains important for preventing pregnancy loss and preterm birth associated with cervical insufficiency [1]. Clinical decision-making is conventionally organized by indication—history-indicated, ultrasound-indicated, or physical examination–indicated cerclage—whereas operations are described mainly by named techniques such as McDonald or Shirodkar [2,3,4]. This vocabulary remains useful, but indication does not fully characterize the cervical state, and a technique name does not establish the reconstruction delivered.

The central proposition of this Perspective is not a new technique, but a shift from procedure-centered cerclage to state-dependent precision cervical reconstruction. Here, **precision cervical reconstruction** denotes a state-informed operative approach linking the observed phenotype and intraoperative state to a defined mechanical objective, an individually selected configuration, and documentation of the reconstruction achieved. “Precision” means explicit correspondence between plan and state; “reconstruction” means restoration of the required mechanical function within available tissue capacity rather than suture placement alone. This is a conceptual reasoning and reporting framework, not a validated treatment-selection algorithm or replacement for established indications and contraindications.

Cervical insufficiency is increasingly understood as a dynamic process involving tissue remodeling, shortening, funneling, dilation, and membrane exposure along variable trajectories [5,6,7,8,9]. Cervical length remains clinically important, but similar dimensions may coexist with different geometries, tissue properties, loading, and rates of progression. Elastography, aspiration-based assessment, and biomechanical modeling provide evidence of such heterogeneity, but variation in equipment, acquisition, sampling, gestational age, and reproducibility precludes their use as independent operative criteria [10,11,12,13].

The ultrasound-indicated phase may represent a decision window before advanced dilation or membrane exposure [14]. Once an emergency operative state develops, patients with similar dilation may differ in membrane-bulging geometry, reducibility, and tissue available for anchoring. Contemporary reviews support examination-indicated cerclage in selected patients while demonstrating substantial heterogeneity in eligibility, co-interventions, and outcomes [15,16]. The B–R–C framework—bulging extent, membrane reducibility, and cervical rim adequacy—describes procedural complexity and feasibility in this setting [17].

A reconstruction is likewise incompletely represented by a procedure name or stitch count. Suture height, axial separation, sequence, material, tissue engagement, and membrane relationship may differ among procedures assigned the same label [18,19,20]. Operator experience, institutional practice, baseline risk, co-interventions, and implementation may further influence outcomes. Operative heterogeneity is therefore one possible contributor to inconsistent findings, not an established explanation.

Four recent author-team publications established separate components rather than the integrated pathway proposed here. The dynamic-process Perspective [5] reframed cervical insufficiency as a temporally evolving condition but did not define an operative-state or reconstruction-reporting architecture. The ultrasound-indicated mini review [14] described a time-dependent decision window but did not connect early phenotype to intraoperative configuration or procedural fidelity. The B–R–C framework [17] standardized complexity and feasibility descriptors for advanced emergency states but did not define mechanical objectives, intended versus achieved reconstruction, or postoperative construct assessment. The historical and technical analysis of modified double-level cerclage [21] clarified operative geometry and sequence but did not provide a cross-indication framework or a minimum dataset linking state, construct, and outcome.

**Figure 1:**
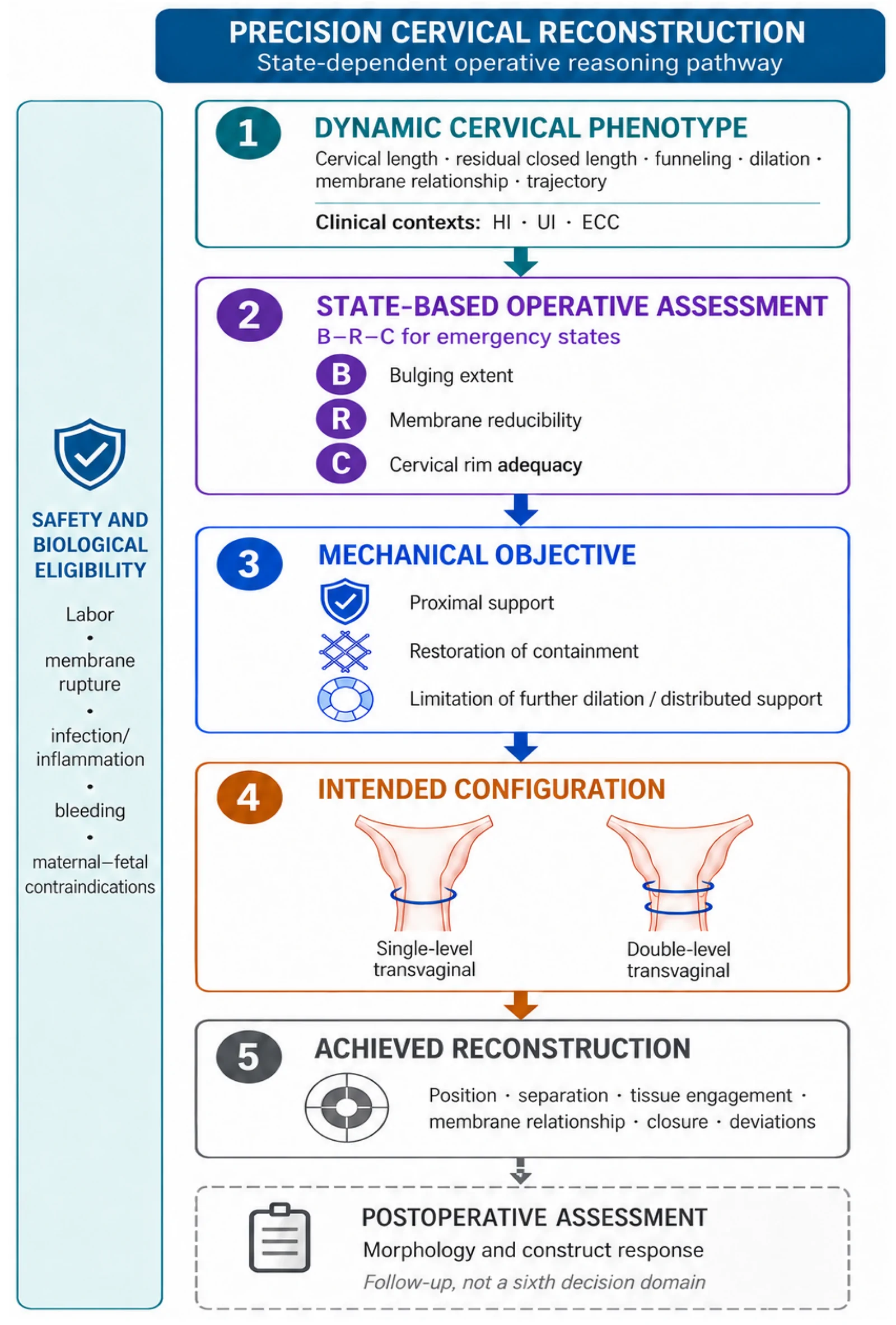
Overall paradigm of precision cervical reconstruction. The framework is presented as a clinical reasoning pathway linking five numbered domains: dynamic cervical phenotype, state-based operative assessment, mechanical objective, intended configuration, and achieved reconstruction. Dynamic cervical phenotype includes cervical morphology and trajectory across history-indicated, ultrasound-indicated, and emergency clinical contexts. In emergency cerclage, the B–R–C framework separately describes membrane bulging extent, membrane reducibility, and cervical rim adequacy. The intended configuration is selected in relation to the stated mechanical objective, available tissue, and procedural safety, whereas achieved reconstruction records what was actually delivered. Safety and biological eligibility—including assessment for labor, membrane rupture, clinically significant infection or inflammation, bleeding, and maternal or fetal contraindications—are cross-cutting constraints that take precedence throughout the pathway. Postoperative assessment describes subsequent morphology and construct response; it is a follow-up component rather than a sixth decision domain. HI, history-indicated cerclage; UI, ultrasound-indicated cerclage; ECC, emergency cervical cerclage; B–R–C, bulging extent–membrane reducibility–cervical rim adequacy.

The present Perspective therefore introduces the relationships among these components rather than claiming each component as new. It integrates five decision domains within a linked pathway—clinical phenotype and trajectory → state-based operative assessment and safety → mechanical objective → intended configuration → achieved reconstruction—followed by postoperative assessment (Figure 1). The sequence applies across history-indicated, ultrasound-indicated, and emergency contexts; B–R–C assessment is most relevant to advanced emergency states rather than a universal second step. Methodologically, the framework separates indication from operative state, stitch count from axial support levels, treatment assignment from procedural fidelity, and planned from delivered geometry. These distinctions enable research applications not developed in the earlier publications: prospective capture of modified, incomplete, or abandoned procedures; prespecified state–configuration interaction analyses; intention-to-treat analyses supplemented by operative-fidelity measures; and cross-center comparison using the minimum state-to-reconstruction dataset in Appendix A. Clinically, the framework structures reasoning, documentation, referral, and interpretation without assigning a technique or claiming validated outcome benefit. Table 1 translates representative findings into possible interpretations and testable objectives while separating clinical observation, biomechanically plausible inference, and research hypothesis. Safety screening—including assessment for labor, membrane rupture, clinically significant infection or inflammation, bleeding, and maternal or fetal contraindications—remains a cross-cutting constraint and may preclude or halt the pathway at any point.

## 2. Approach to Evidence Identification and Conceptual Synthesis

This Perspective was informed by a structured narrative assessment of clinical and mechanistic literature. PubMed/MEDLINE was searched from database inception through 8 September 2026 using combinations of “cervical insufficiency,” “cervical cerclage,” “history-indicated,” “ultrasound-indicated,” “physical examination-indicated,” “emergency cerclage,” “cervical remodeling,” “biomechanics,” “elastography,” “suture height,” “cerclage position,” “single stitch,” “two stitches,” “double cerclage,” and “double-level cerclage.” Reference lists of relevant guidelines, systematic reviews, randomized trials, and mechanistic studies were also examined.

Sources were selected for their role in the framework rather than through an exhaustive inclusion process. Priority was given to current guidance, systematic reviews, randomized trials, studies directly evaluating operative configuration or postoperative geometry, and mechanistic or imaging studies relevant to tissue properties and support. Recent independent sources were preferred; older studies were retained only when seminal or directly comparative. Clinical evidence, plausible mechanical interpretation, and hypotheses requiring validation were treated as distinct categories.

The assessment was intended to define conceptual boundaries and locate the strongest available evidence for each component, rather than to estimate a pooled treatment effect. Guidance and clinical reviews informed established eligibility and safety principles; comparative studies informed claims about technique and suture number; imaging and biomechanical studies informed only mechanistic plausibility and measurement priorities. Where a recent publication addressed the same question as an older source, the recent independent source was preferred unless the earlier report contained foundational or uniquely comparative information.

This is not a systematic review. No formal risk-of-bias assessment, quantitative synthesis, or certainty grading was performed. ChatGPT (GPT-5, OpenAI, San Francisco, CA, USA; accessed August–September 2026) assisted language refinement, structural organization, condensation, and development of candidate search terms. The authors performed literature selection, bibliographic verification, interpretation, and final scientific review and take responsibility for the manuscript.

## 3. Why the Existing Paradigm Is Incomplete

Indication-based classification, cervical measurement, and named techniques remain clinically useful, but none alone describes the complete cervical state, operative task, or reconstruction delivered.

### 3.1. Indication and Measurement

History-indicated, ultrasound-indicated, and physical examination–indicated cerclage describe the pathway to intervention [2,3,4]. Each encompasses heterogeneous states: similar cervical lengths may accompany different funnel geometries, residual closed lengths, tissue characteristics, or trajectories, while similar dilation may coexist with different membrane exposure, reducibility, and anchoring tissue. Indication establishes context; phenotype and **state-based operative assessment** describe the condition encountered.

Shortening and dilation are observable manifestations of deformation, not direct measures of stiffness, load-bearing or suture-holding capacity, or tissue response to compression [6,7,8,9,10,11,12,13]. Emerging biomechanical methods may refine characterization but have not been shown to improve patient selection. They remain complementary research tools rather than substitutes for validated criteria.

This distinction matters because an anatomical measurement and a mechanical interpretation answer different questions. Cervical length describes geometry at a specified examination; it does not reveal how the remaining tissue will respond to increasing uterine load, operative traction, circumferential compression, or continued remodeling. Likewise, dilation describes an advanced manifestation but does not identify whether membranes can be reduced or whether a continuous rim can safely retain a suture. Measurement should therefore remain central to diagnosis and surveillance while being embedded in a broader description of morphology, trajectory, membrane relationship, and operative findings.

### 3.2. Procedure Labels and Comparative Evidence

McDonald, Shirodkar, and stitch count do not reliably specify proximal height, axial separation, sequence, circumferential engagement, membrane position during tightening, or postoperative geometry [18,19,20]. Two sutures may reinforce one axial zone or occupy distinct levels. Named technique, intended configuration, and achieved reconstruction are therefore separate descriptors.

Comparative studies estimate average effects of assigned strategies. Differences may reflect sample size, baseline risk, patient selection, gestational age, co-interventions, endpoints, operator experience, center practice, and implementation. Cervical state and achieved configuration may contribute but should not be presumed to explain conflicting results. State–configuration interaction is a testable hypothesis: effectiveness studies determine whether assignment improves outcomes, whereas mechanistic studies determine what was delivered and how it interacted with tissue and geometry.

## 4. From Dynamic Phenotype to Operative State

### 4.1. The Cervix as a Dynamic Mechanobiologic System

The cervix maintains pregnancy through interactions among tissue composition, geometry, and pregnancy-related loading. Remodeling alters collagen organization, hydration, stiffness, viscoelastic behavior, and resistance to deformation, while uterine growth changes loads transmitted toward the internal os [6,7,8,9]. Competence is therefore evolving rather than fixed.

Length, funneling, dilation, membrane position, rim continuity, attenuation, friability, and deformation during traction are observable findings. “Tissue competence,” by contrast, is an inferred property encompassing the ability to resist loading, retain a suture, and tolerate compression. Observation should be documented separately from mechanical interpretation.

**Figure 2:**
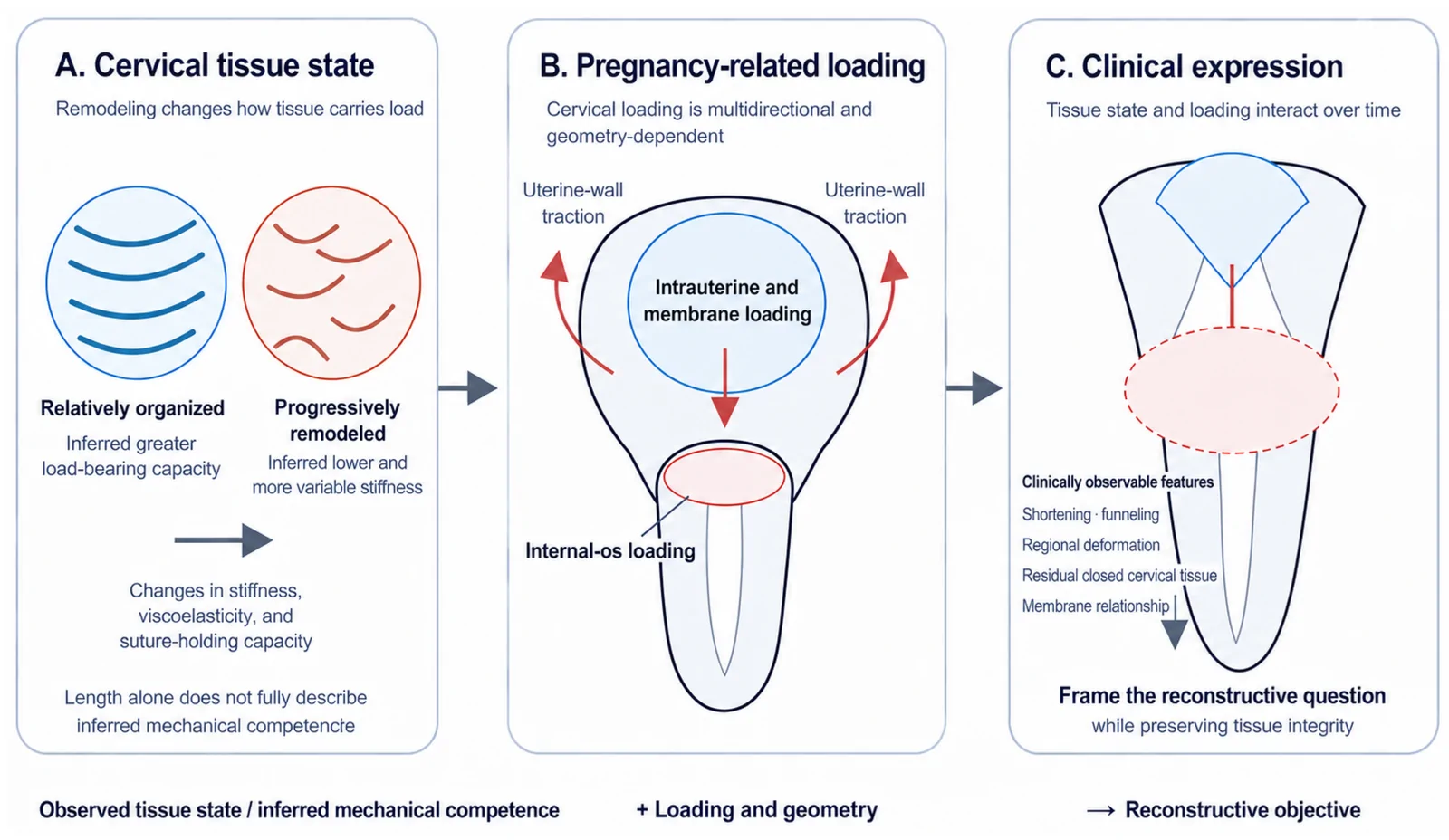
Multiscale determinants of inferred cervical mechanical competence and the reconstructive question. This conceptual diagram does not depict measured patient-specific force or stress distributions and does not provide validated thresholds for selecting a cerclage configuration. (**A**) Pregnancy-related remodeling changes tissue organization, stiffness, viscoelasticity, load-bearing behavior, and suture-holding capacity. Tissue state is observable only through its clinical manifestations and research measurements; mechanical competence remains an inferred property. (**B**) Cervical loading is multidirectional and geometry-dependent and may include uterine-wall traction and intrauterine or membrane loading transmitted toward the internal os. (**C**) The interaction between tissue state and loading evolves over time and may be expressed clinically as shortening, funneling, regional deformation, variation in residual closed cervical tissue, and changes in membrane relationship. These observations frame the reconstructive question while preserving tissue integrity. Elastography, aspiration-based measurements, and biomechanical modeling remain research-oriented and should not currently determine cerclage indication, suture number, placement height, or postoperative intervention.

Cerclage introduces circumferential support into living, remodeling tissue. Its effect depends on position, depth, engagement, tension, material, and relationship to the internal os and membranes [18,19,20]. Compression may initially alter deformation, while later remodeling, migration, tissue cutting, or recurrent funneling may change the construct. Figure 2 therefore represents conceptual relationships among tissue, loading, geometry, and support; it does not depict measured patient-specific forces.

The operative objective is consequently functional rather than purely procedural. Depending on the state encountered, the required function may include reinforcing a proximal region, restoring containment after membrane reduction, limiting further dilation, or distributing support across more than one axial level. These are proposed operative objectives, not independently validated indications. A technically complete operation may fail to reproduce the geometry intended before surgery, whereas a less extensive construct may provide the required function when proximal placement and tissue engagement are favorable. The relevant question is not how much suture material was placed, but what support was safely achieved within the tissue available and how that construct behaved over time.

### 4.2. The Ultrasound-Indicated Window

The ultrasound-indicated phase lies between risk defined mainly by history and an advanced state characterized by dilation or membrane exposure [14]. Progressive shortening, funneling, or loss of residual closed cervix may indicate evolving failure, but these findings do not follow a uniform sequence. History-, ultrasound-, and emergency indications are therefore detection and intervention contexts rather than fixed biological stages.

An ultrasound-indicated case with measurable proximal tissue and confined membranes presents a different task from an emergency case requiring membrane reduction. Elastography may eventually complement morphology but should not currently determine indication, suture number, or height [10,11,12,13].

For operative description, cervical length should therefore be interpreted together with residual closed length, funnel configuration, internal-os change, regional attenuation, membrane relationship, and serial trajectory when available. These features do not create a new indication for cerclage. They describe the state in which an established indication is being considered and may clarify why apparently similar cases present different reconstructive constraints. The transition between contexts is also not necessarily linear: rapid change may occur between examinations, while some short cervices remain morphologically stable. The framework is designed to record such differences without converting them into unvalidated treatment thresholds.

### 4.3. Emergency Operative State and Safety

With dilation and exposed or prolapsed membranes, planning expands from whether cerclage is indicated to whether reconstruction is safe and technically feasible. Assessment should address contractions, preterm prelabor rupture of membranes, clinically significant intra-amniotic infection or inflammation, bleeding, fetal status, gestational age, and maternal or fetal contraindications [2,4,15,16]. Safety and biological eligibility are cross-cutting constraints that take precedence over procedural ambition; technical feasibility does not establish clinical appropriateness.

The B–R–C framework separates three dimensions commonly compressed into dilation alone [17]:

1. **Bulging extent (B):** B1, membranes confined within the endocervical canal; B2, membranes reaching the external os; B3, membranes protruding beyond the external os; and B4, advanced prolapse extending deeply into the vagina. Bulging extent describes the anatomical position of the membranes and modifies procedural complexity but does not independently determine feasibility.
2. **Membrane reducibility (R):** R1, membranes reducing spontaneously or with minimal manipulation; R2, reduction achieved with adjunctive measures; R3, partial or unstable reduction; and R4, membranes irreducible with standard reduction attempts. Reducibility reflects the feasibility and stability of membrane repositioning and should be documented at the time assessed.
3. **Cervical rim adequacy (C):** C1, a thick, circumferential rim suitable for secure anchoring; C2, a mildly reduced but continuous rim; C3, a thin or asymmetrical rim with limited tissue support; and C4, a severely effaced or discontinuous rim for which anchoring is not reliable. Cervical rim adequacy describes the amount, continuity, and apparent structural integrity of the residual tissue available for suture anchoring rather than proven long-term mechanical capacity.

B–R–C describes operative feasibility and procedural complexity, not prognosis or a mandated technique. Reducibility and cervical rim adequacy are the primary determinants of technical feasibility, whereas bulging extent modifies procedural complexity once feasibility has been established [17]. An R4 or C4 state indicates technically non-feasible ECC within the framework; R3 and/or C3 identifies high-complexity but potentially feasible ECC. Timing, reduction method, anesthesia, instrumentation, operator experience, and institutional capability may influence assessment. Standardized training, reliability testing, and multicenter validation remain necessary. Because the operative state may change between presentation and the procedure, both the initial state and the state immediately before or during cerclage should be documented.

The transition from phenotype to operative state can be framed by three questions: What failure is observable? What function may need to be restored? What reconstruction, if any, can be achieved safely within the available tissue? The answers may change during preparation. Membrane reduction may improve visualization and reveal a usable cervical rim, whereas subsequent manipulation may reveal unexpected tissue fragility or inadequate purchase. Recording both the initial and updated state avoids treating operability as a fixed preoperative attribute and distinguishes the technical question of what can be constructed from the clinical judgment of whether proceeding remains appropriate.

## 5. Precision Cervical Reconstruction

### 5.1. Mechanical Objective and Operational Terms

Precision cervical reconstruction begins by defining the function to be restored: proximal reinforcement, membrane containment, limitation of further dilation, preservation of residual tissue, or support across more than one axial level. These objectives are not new indications.

**Proximal support** denotes circumferential reinforcement placed as close to the functionally relevant proximal region as can be achieved safely, without implying a universal distance from the internal os. **Restoration of containment** denotes reduction of exposed or prolapsed membranes above the intended reconstruction and closure without evident membrane interposition; it is an intraoperative description, not proof of pregnancy maintenance. **Distributed support requirement** denotes a hypothesized need for more than one axial support region; no validated clinical criterion establishes it. Meaningful vertical separation denotes retained circumferential support at anatomically distinct proximal and distal levels, creating a longer reconstructed segment. **Adequate tissue engagement** denotes observable incorporation sufficient to retain the suture without immediate pull-through, tearing, or membrane entrapment; it does not prove long-term competence.

### 5.2. Tissue-Constrained Configuration

The selected construct should be the least extensive configuration reasonably capable of achieving the intended function within available tissue. Favorable proximal tissue may permit effective single-level support. Residual length with two usable regions may permit separated reinforcement when broader support is intended. Attenuated, friable, or marginal tissue may limit height, bite depth, tension, or suture number.

Configuration includes the named technique, number of sutures, number of intended axial support levels, position and function of each suture, placement sequence, tissue engagement, material, caliber, and applied tension. A simplified mechanical model suggests that higher placement, greater thread caliber, and more sutures may increase theoretical confining force, but these relationships do not establish clinical superiority and may be limited by tissue tolerance and model assumptions [22]. Material structure and caliber can affect handling, knot security, friction at the tissue–suture interface, distribution of compressive contact, and the potential for tissue cutting or pull-through. Braided sutures or tape and monofilament sutures also differ in surface characteristics, tissue interaction, microbial adherence, visibility, and ease of removal; however, these properties interact with tension, bite depth, tissue quality, construct geometry, and operator technique. Comparative clinical evidence has not established a universally superior material for every cervical state or objective [23]. Material and caliber should therefore be documented as operative descriptors and studied as potential modifiers rather than treated as unqualified markers of quality.

Additional sutures, higher placement, or prolonged manipulation may increase membrane rupture, bleeding, laceration, tissue cutting or ischemia, infection, distortion, migration, operative time, technical failure, and removal difficulty. A more extensive construct is not intrinsically more precise. Plans should be limited, modified, or abandoned when safety or tissue tolerance is uncertain. Operator and center factors should be treated as potential confounders or effect modifiers because they influence feasibility, achieved geometry, complications, and outcomes.

Planning can be organized as a conditional sequence rather than a technique hierarchy. First, define the mechanical deficit suggested by the observed state. Second, identify tissue that can be engaged without unacceptable membrane or tissue risk. Third, select the least extensive construct reasonably capable of meeting the objective. Fourth, verify during placement whether the intended geometry remains achievable. Finally, document what was retained and why it differed from the plan, if it did. This sequence is deliberately nonprescriptive: it structures reasoning and reporting but supplies neither a score nor a rule assigning a particular configuration.

### 5.3. Intended and Achieved Reconstruction

Intent and delivery should be documented separately. A planned proximal suture may be placed lower because of exposure, membrane position, or tissue limitation; planned levels may approximate after tightening; and a distal suture may function primarily as closure rather than reinforcement. Conversely, favorable exposure may permit more proximal support than anticipated.

The intended configuration should specify the mechanical objective, planned number of sutures, planned number of axial support levels, intended position and function of each suture, intended inter-level separation, placement sequence, material, and caliber. The achieved reconstruction should record the number of sutures placed and retained, number of axial support levels achieved, position and function of each suture, measured or described inter-level separation, tissue engagement, membrane position during tightening, completeness of closure, tissue response, complications, and deviations from the intended configuration. Table 1 translates findings into possible interpretations and objectives while distinguishing clinical observation, biomechanically plausible inference, and research hypothesis.

Postoperative assessment is related to, but distinct from, achieved reconstruction. The operative record captures what the surgeon observed and delivered at the time of placement, whereas subsequent imaging captures morphology after tissue deformation, uterine loading, healing, and remodeling have resumed. Agreement among the intended configuration, operative record, and later imaging cannot be assumed. Keeping these stages separate permits investigation of procedural fidelity and temporal change without implying that a postoperative image alone determines whether the original mechanical objective was achieved.

## 6. Localized Versus Vertically Distributed Reinforcement

### 6.1. Distinct Geometries

Three common configurations should be distinguished: (1) conventional single-level cerclage, comprising one circumferential suture at a single cervical level; (2) same-level double-stitch reinforcement, comprising two sutures placed at the same or closely adjacent cervical level; and (3) double-level reconstruction, comprising two retained circumferential sutures placed at anatomically distinct proximal and distal levels [21]. “Two-stitch” should be used when suture number is known but vertical separation is not confirmed, whereas “double-level” should require intended and documented placement at separate cervical levels.

Suture number and support-level number should nevertheless be reported separately because more complex configurations may occur. For example, two sutures reinforcing one proximal level together with an additional distal suture constitute a three-suture, two-level configuration rather than a distinct category of “triple cerclage.” This distinction may be particularly relevant in repeat or revision cerclage, in which a retained or reinforced support level may coexist with an additional suture placed at another level. Such configurations are descriptive possibilities, not validated treatment strategies. The intended function of each suture—structural support, supplemental reinforcement, limitation of distal dilation, or closure—should therefore be specified.

### 6.2. Conditional Mechanical Hypothesis

A single support level localizes circumferential reinforcement, whereas vertically separated retained levels might extend the supported cervical segment, provide structural redundancy, or alter regional deformation. A simplified mechanical model suggests that higher placement, greater thread caliber, and a greater number of sutures may increase theoretical confining force [22]. However, that model does not compare vertically separated operative constructs or establish clinical benefit. The proposed effects of distributed support therefore remain biomechanically plausible hypotheses rather than validated biomechanical or clinical effects.

Any incremental effect would be expected to depend on cervical state, available anchoring tissue, proximal position, maintained inter-level separation, and the intended function of each suture. An additional suture may add little when it reinforces approximately the same axial region, when one level already restores the required function, or when intended separation collapses during tightening. Even when multiple sutures are retained, their functions may differ: a proximal suture may provide the principal structural support, whereas an additional suture may reinforce the same level, supplement distal closure, or constitute an independent support level. Stitch count alone cannot distinguish these configurations.

The potential benefit of creating an additional support level must be weighed against its procedural cost. In a short, attenuated, friable, or irregular cervix, additional manipulation may leave insufficient tissue for secure engagement at each intended level or increase bleeding, laceration, membrane rupture, tissue cutting, ischemia, distortion, migration, infection, operative time, technical failure, or difficulty at removal. Distributed support should therefore be considered only within the limits of available tissue and procedural safety, not as an inherently preferable configuration.

**Figure 3:**
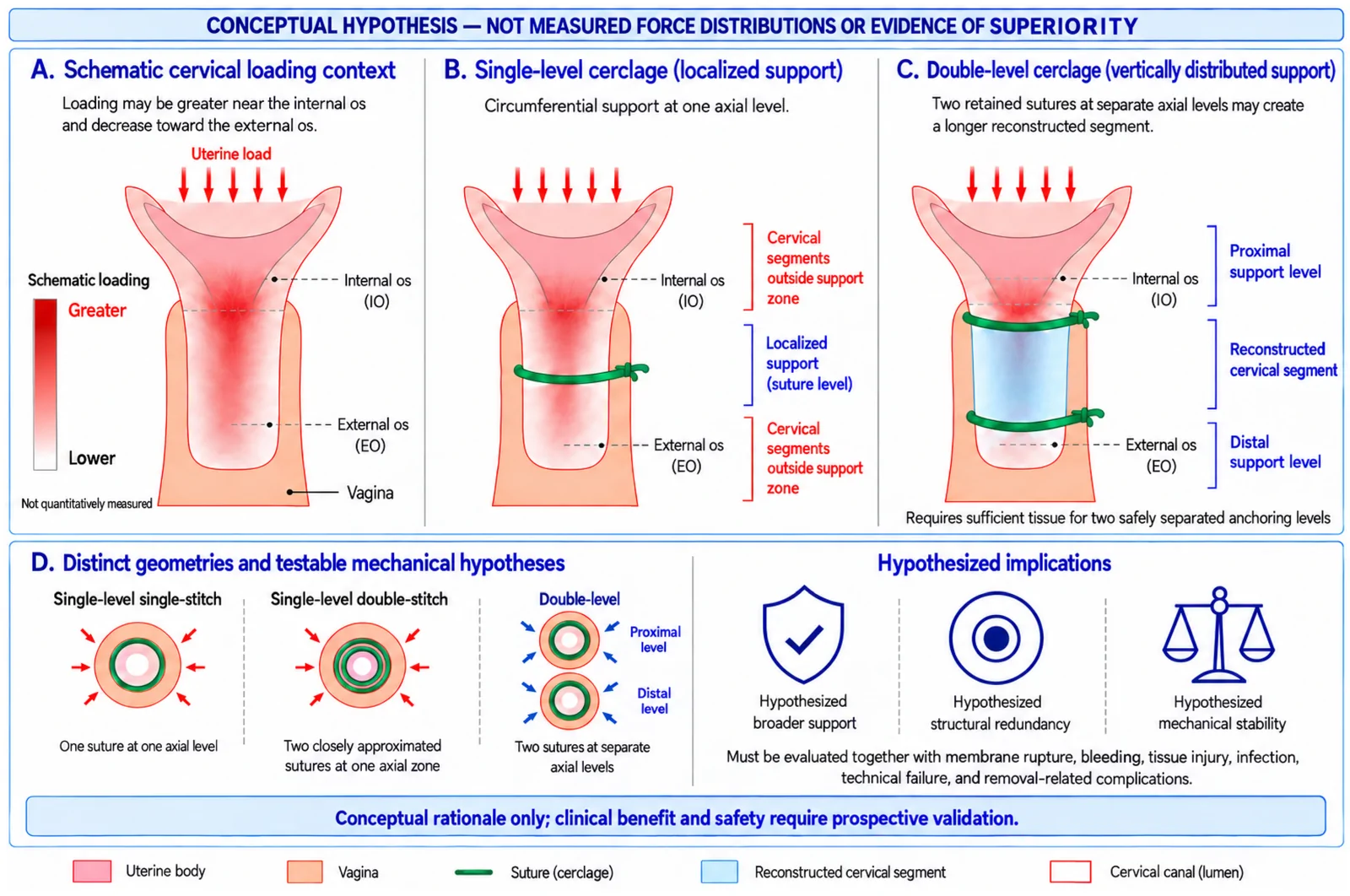
Conceptual mechanical rationale for localized and vertically distributed cervical support. This figure presents a conceptual hypothesis; it does not depict measured force distributions or establish biomechanical or clinical superiority of any configuration. (**A**) A schematic loading context illustrates the hypothesis that pregnancy-related loading may be greater near the internal os and decrease toward the external os; the gradient is not quantitatively measured. (**B**) A single-level cerclage provides localized circumferential support at one axial level, leaving cervical segments above and below the supported zone. (**C**) Two retained sutures placed at safely separated proximal and distal levels may create a longer reconstructed cervical segment and vertically distributed support, provided that sufficient tissue is available for both anchoring levels. (**D**) The geometry of single-level single-stitch, single-level double-stitch, and double-level reconstruction should be distinguished: two closely approximated sutures at one axial zone do not constitute double-level reconstruction. Broader support, structural redundancy, and mechanical stability are testable hypotheses rather than established effects. Potential benefits must be evaluated together with membrane rupture, bleeding, tissue injury, infection, technical failure, and removal-related complications. Clinical benefit and safety require prospective validation. Red arrows indicate the hypothesized direction and relative gradient of pregnancy-related loading, whereas blue arrows indicate intended circumferential support at the represented suture levels. IO, internal os; EO, external os.

Figure 3 illustrates operative geometry, not measured force distribution or superiority. Prospective studies should test whether intended levels remain measurably separated, whether separated constructs produce postoperative geometry distinct from same-level reinforcement, and whether any effect varies with cervical state, tissue availability, proximal position, and the stated function of each suture. Morphologic differences should not be considered clinically meaningful unless they are associated with pregnancy latency, gestational age at delivery, maternal morbidity, and neonatal outcomes without disproportionate complications. These are testable predictions, not a recommendation for routine double-level cerclage.

## 7. Interpreting the Existing Evidence

### 7.1. Earlier Evidence

Earlier comparisons of one- and two-suture cerclage were heterogeneous across clinical indications and did not consistently distinguish same-level reinforcement from vertically separated double-level support [21]. In history-indicated settings, a consistent advantage of two sutures has not been established, although one randomized trial in women with previous second-trimester loss reported later delivery and higher birth weight with double cerclage [24]. Evidence specific to ultrasound-indicated cerclage remains limited and does not establish a consistent benefit. In more advanced states requiring emergency cerclage, a small retrospective study reported higher gestational age at delivery and fewer early preterm births with two stitches [25]. An independent systematic review likewise found the available comparative evidence to be heterogeneous and insufficient to establish the overall superiority of double over single cerclage, although a potential benefit in selected clinical settings could not be excluded [26].

Viewed alongside the mechanical rationale for distributing support across more than one axial level, these uneven findings raise—but do not establish—the hypothesis that the incremental value of vertically distributed support may become more clinically apparent as cervical deformation advances. This proposed state-dependent gradient remains hypothesis-generating because the available studies did not isolate stitch number from vertical separation, suture height, tissue engagement, patient selection, or co-interventions. Accordingly, the evidence does not support routine double cerclage across all indications. Rather, it identifies a testable possibility that an additional, vertically separated support level may be most relevant in advanced cervical states, in which deformation is more extensive and a single localized support zone may be insufficient.

### 7.2. Recent Randomized Evidence

Randomized evaluation in emergency cervical cerclage is particularly challenging because the operative state may evolve rapidly and technical feasibility may change between enrollment and the procedure [17]. Multicenter trials must additionally accommodate individualized safety decisions while standardizing assessment and implementation across operators and institutions. Despite these challenges, two recent randomized studies have provided evidence relevant to emergency cerclage [27,28].

The Chinese stratified exploratory trial compared one- and two-stitch McDonald cerclage in ultrasound-indicated and emergency strata [27]. One experienced operator used the same material; the proximal suture in the two-stitch group was followed by a distal suture approximately 1–1.5 cm below it, partly to close the external os. The primary outcome of spontaneous preterm birth before 34 weeks did not differ significantly within either stratum. In the emergency subgroup, birth before 28 weeks occurred in 12.0% versus 40.0% (RR, 0.30; 95% CI, 0.09–0.96; *p* = 0.024). Because this was a small stratum-specific comparison with a nonsignificant primary outcome, possible multiplicity, and a wide confidence interval, the finding is hypothesis-generating and does not validate the proposed framework.

The Polish multicenter trial compared assigned single-level and double-level emergency cerclage strategies across seven tertiary centers [28]. Delivery before 34 weeks occurred in 31.0% and 23.7%, respectively (*p* = 0.527), without statistically significant differences in gestational age at delivery, latency, other preterm-birth thresholds, or neonatal outcomes. The trial therefore did not demonstrate clinical superiority of the tested double-level strategy. Plausible interpretations include no clinically important advantage, insufficient statistical precision, heterogeneity in baseline risk or operative state, implementation variation, and the selected endpoint or anticipated effect size. These alternatives should be considered without presuming that procedural heterogeneity explains the neutral result.

### 7.3. Assignment, Fidelity, and Interpretation

Randomization estimates the effect of assignment, whereas operative-fidelity data characterize the reconstruction actually delivered. Incomplete procedural reporting does not invalidate intention-to-treat analysis; it limits only construct-specific mechanistic interpretation. The Chinese study standardized the operator and intended inter-suture interval but did not prespecify postoperative verification of maintained functional separation [27]. The Polish trial did not systematically characterize achieved suture height, separation, tissue engagement, or postoperative geometry [28]. Its neutral result remains clinically informative for the assigned strategies as implemented, while leaving unresolved whether outcomes varied with achieved reconstruction.

Current evidence establishes neither universal superiority of double-level cerclage nor a general requirement for an additional suture. Single-level reconstruction may adequately restore the intended function in some states, whereas an additional suture may not provide incremental benefit when it reinforces the same axial region or when the required function has already been restored. Vertically distributed reinforcement remains plausible but unproven when broader support is intended and available tissue permits separate levels. A state–configuration interaction should therefore be tested prospectively rather than imposed retrospectively on neutral trial results.

The evidence hierarchy must remain explicit. Randomized clinical outcomes take precedence when evaluating comparative benefit. Mechanistic plausibility can motivate measurement and trial design but cannot reverse a neutral clinical comparison. Operative-fidelity data may clarify whether the intended contrast was delivered but should not be used post hoc to manufacture efficacy. Observational state–outcome associations may generate hypotheses, but they remain vulnerable to selection, operator, center, and co-intervention effects. Future randomized studies should retain intention-to-treat analysis as the primary estimate, prespecify the intended mechanical contrast, and document operative fidelity without retrospectively redefining the trial. Prespecified secondary analyses may examine whether intended configurations were achieved, whether operative state modified the effect of assignment, and whether measured geometric differences were associated with clinical outcomes. Such analyses require reliable definitions and adequate sample size, and exploratory findings should not become clinical recommendations without independent confirmation.

## 8. Reporting and Validation

### 8.1. Minimum State-to-Reconstruction Dataset

The five domains in Figure 1 translate into five core reporting components, with safety and biological eligibility incorporated as cross-cutting constraints; a corresponding practical form is provided in Appendix A. Postoperative assessment records subsequent morphology and response to the construct rather than constituting a sixth decision domain.

1. **Dynamic phenotype:** Indication, gestational age, relevant obstetric history and treatment, cervical length and residual closed length when measurable, dilation, funnel configuration, membrane relationship, and serial trajectory.
2. **Operative state and safety eligibility:** Contractions, membrane status, assessment for clinically significant infection or inflammation, bleeding, fetal and maternal status, contraindications, and B–R–C descriptors when applicable. The method and result of membrane reduction and any transition in operative state during preparation should be recorded [17].
3. **Mechanical objective:** Intended proximal support, containment, limitation of further dilation, preservation of residual tissue, distal closure, or distributed reinforcement.
4. **Intended configuration:** Named technique; planned number of sutures; planned number of axial support levels; intended position and function of each suture; intended inter-level separation; placement sequence; material and caliber; and the planned management of any pre-existing retained suture.
5. **Achieved reconstruction:** Number of sutures placed and retained; number of axial support levels achieved; position and function of each suture; measured or described inter-level separation; tissue engagement; membrane position during tightening; completeness of closure; tissue response; status of any pre-existing suture; complications; and deviations from the intended configuration.

Operator experience and institutional context should be reported because they may affect feasibility, procedural fidelity, complications, and outcomes. The dataset standardizes description; it does not prescribe whether cerclage should be performed or which configuration should be selected.

### 8.2. Postoperative Assessment

Ultrasonography can describe suture position, residual closed length, funnel morphology, membrane relationship, and the relative positions of multiple sutures [18,19,20]. However, published studies vary in imaging route, timing, measurement plane, landmarks, and reported outcomes. No universal protocol establishes the preferred use of transvaginal or transabdominal imaging, optimal assessment interval, reference point for suture height, method for measuring inter-suture or inter-level separation, or threshold for changing management. In routine practice, ultrasonography may therefore be used according to clinical context to assess conventional cervical morphology, membrane position, residual closed length, or suspected complications. Exact suture height, maintained inter-level separation, tissue engagement, and serial geometric change should remain research variables requiring standardized acquisition and reliability assessment.

Research protocols should prespecify imaging route, timing, bladder status, plane, landmarks, repeated measurements, operator training, and assessment of intra- and interobserver reliability. Elastography may characterize regional responses around or between sutures, but postoperative differences may reflect compression, remodeling, adaptation, measurement artifact, or combinations of these factors [10,11,12,13]. Device and software dependence, probe pressure, sampling region, gestational age, and observer variability preclude its current use for determining cerclage indication, suture number, support-level number, suture height, or postoperative intervention. A technically measurable variable is not necessarily clinically actionable, and no postoperative geometric or elastographic threshold is currently validated for changing management.

### 8.3. Illustrative Documentation Pathways

These examples illustrate documentation pathways, not treatment recommendations.

In a history-indicated case, documentation would include the relevant obstetric and operative history, current cervical phenotype, safety eligibility, and the mechanical objective of the planned procedure. The intended number of sutures, number of axial support levels, position, sequence, material, and function of each suture would be distinguished from the configuration ultimately achieved, including any deviation from the plan.

In an ultrasound-indicated case with progressive shortening and funneling, measurable residual closed cervix, and confined membranes, documentation would proceed from phenotype, trajectory, and safety eligibility to the intended mechanical objective and planned configuration. The operative record would distinguish the planned number of sutures and support levels from the positions, tissue engagement, material, closure, and reconstruction actually achieved. Postoperative morphology would be assessed according to local clinical practice, whereas quantitative geometry would require a prespecified research protocol.

In an emergency case with dilation and prolapsed membranes, documentation would additionally include biological eligibility, the initial and updated B–R–C state, the membrane-reduction method and result, and the cervical tissue available for anchoring [17]. The intended objective and selected configuration would be distinguished from the reconstruction ultimately achieved, including the number of sutures retained, number of axial support levels achieved, position and function of each suture, maintained separation, membrane position during tightening, tissue response, complications, and reasons for modification or discontinuation. Technical feasibility and prognosis remain distinct judgments.

### 8.4. Staged Validation and Clinical Implications

Validation should proceed sequentially. Initial studies should standardize terminology, anatomical landmarks, imaging planes, and measurement protocols; establish the feasibility of data collection and intra- and interobserver reliability; and determine whether B–R–C grades, tissue descriptors, mechanical objectives, intended configurations, and achieved-reconstruction variables can be recorded consistently across operators and centers. Subsequent prospective studies should assess associations with procedural complications, pregnancy latency, gestational age at delivery, preterm prelabor rupture of membranes, maternal morbidity, and neonatal outcomes. Only after these foundations are established should comparative studies test whether state-guided reconstruction improves clinical outcomes. Randomized trials should retain intention-to-treat analysis as the primary estimate, prespecify the intended mechanical contrast, document operative fidelity, and report harms alongside benefits. State-enriched trials may increase sensitivity to conditional effects, although narrower eligibility may reduce generalizability.

The framework’s immediate clinical value lies in explicit reasoning, documentation, referral, and intercenter communication rather than validated treatment selection. It separates indication from operative state, technical feasibility from clinical appropriateness, and intended configuration from achieved reconstruction. It also makes visible procedures that are modified, deferred, transferred, attempted but not completed, or not attempted. Such cases may define the feasibility boundary yet disappear from cohorts restricted to completed procedures. Standardized description, reproducible measurement, and transparent reporting of these pathways should precede claims of configuration-specific superiority.

The framework primarily concerns transvaginal reconstruction during pregnancy. Transabdominal cerclage (TAC) is a separate escalation pathway that may be considered after an appropriately characterized failed transvaginal cerclage, when the cervix is severely shortened or structurally damaged, or when anatomy precludes adequate transvaginal placement. TAC may be performed before conception or, in selected patients, during pregnancy; timing requires individualized consideration of obstetric and operative history, current pregnancy status, anatomy, surgical access, and maternal–fetal risk. It should not be treated as another configuration within the present transvaginal framework. In history-indicated and ultrasound-indicated contexts, the framework integrates prior operative history, morphology, and trajectory while withholding treatment-selection authority from unvalidated biomechanical measurements. In emergency cerclage, it separates anatomical severity, procedural complexity, technical feasibility, and prognosis. Extensive bulging may increase complexity but does not independently establish technical non-feasibility; membrane reducibility, cervical rim adequacy, biological eligibility, safety constraints, operator capability, and institutional resources more directly determine whether reconstruction can be attempted [17].

Precision lies not in procedural complexity but in correspondence among state, objective, tissue capacity, safety, and documented achievement. In emergency cerclage, surgical skill is essential, but timing also has ethical significance: responsible care includes recognizing deterioration before technical rescue becomes the only remaining measure of action. The minimum reporting principle is: **Report the state encountered, the safety constraints assessed, the mechanical objective intended, the configuration planned, and the reconstruction achieved**.

## 9. Conclusions

Precision cervical reconstruction reframes cerclage from selection of a named procedure toward explicit linkage of clinical phenotype and operative state with a defined mechanical objective, intended configuration, and achieved reconstruction, within the constraints of tissue capacity and safety. It neither replaces established indications nor constitutes a validated treatment-selection algorithm.

The framework distinguishes clinical observation from biomechanically plausible inference and testable research hypothesis. Double-level reconstruction is one conditional configuration rather than a universally preferred strategy. Current evidence establishes neither its general superiority over single-level cerclage nor a validated cervical state in which distributed support improves clinical outcomes.

Its immediate contribution is a structured approach to clinical reasoning, operative documentation, and research comparability. Future studies should standardize terminology and measurement, establish reliability, document operative fidelity and harms, and relate intended configuration and achieved reconstruction to maternal and neonatal outcomes. The proposed shift is from naming a procedure for an indication to documenting what mechanical function was intended, what configuration was planned, and what reconstruction was safely achieved in the state encountered.

## Figures and Tables

**Table 1:** State-Dependent Translation from Cervical Findings to Reconstructive Objectives.

Framework Domain	Representative Information Documented	Interpretive Role	Objective or Documentation Output	Evidentiary Status
**Dynamic cervical phenotype**	Indication and gestational age; cervical length and residual closed length when measurable; dilation; funnel configuration; membrane relationship; serial trajectory	Describes the observable cervical state and its evolution without treating indication as a biological stage	State description used to frame the subsequent operative assessment	Established clinical observations; their configuration-specific implications require validation
**Operative state and safety eligibility**	Contractions; membrane status; clinically significant infection or inflammation; bleeding; fetal and maternal status; contraindications; B–R–C descriptors when applicable; method and result of membrane reduction; change during preparation	Separates biological eligibility and safety from procedural complexity and technical feasibility	Document whether reconstruction is clinically appropriate and technically feasible in the state encountered	Safety assessment reflects established clinical principles; B–R–C reproducibility and prognostic relevance require prospective validation
**Mechanical objective**	Intended proximal support; restoration of containment; limitation of further dilation; preservation of residual tissue; distal closure; or support across more than one axial level	States the function the reconstruction is intended to restore, rather than naming a procedure alone	Explicit, prespecified operative objective	Biomechanically plausible interpretation; not an independently validated indication or treatment rule
**Intended configuration**	Named technique; planned number of sutures; planned number of axial support levels; intended position and function of each suture; intended inter-level separation	Translates the objective into a tissue- and safety-constrained operative plan	Record the planned configuration and the reasons for selecting, limiting, modifying, or abandoning it	State-dependent operative plan; superiority of any configuration is not established
**Achieved reconstruction**	Number of sutures placed and retained; number of axial support levels achieved; position and function of each suture; measured or described inter-level separation; circumferential tissue engagement; membrane position during tightening; completeness of closure; tissue response; complications; deviations from plan	Distinguishes the reconstruction delivered from the technique assigned or intended	Direct operative record for assessment of procedural fidelity and subsequent postoperative response	Direct clinical observation; associations with maternal and neonatal outcomes require validation

Abbreviations: B–R–C, bulging extent–membrane reducibility–cervical rim adequacy. Note: Safety and biological eligibility are cross-cutting constraints rather than a sixth domain. The table organizes clinical description, operative documentation, and hypothesis generation; it does not prescribe whether cerclage should be performed or which configuration should be selected. Postoperative assessment records the subsequent response to the construct and is not a sixth decision domain.

## Data Availability

No new data were created or analyzed in this study. Data sharing is not applicable to this article.

## Abbreviations

HI	History-indicated cerclage
UI	Ultrasound-indicated cerclage
ECC	Emergency cervical cerclage
B–R–C	Bulging extent–membrane reducibility–cervical rim adequacy
TAC	Transabdominal cerclage

## Appendix A. Minimum State-to-Reconstruction Reporting Form

**Table A1:** Minimum State-to-Reconstruction Reporting Form. This form standardizes description and does not prescribe whether cerclage should be performed or which configuration should be selected.

Reporting Domain	Items to Document
Dynamic cervical phenotype	Indication and gestational age; relevant obstetric history and treatment; cervical length and residual closed length when measurable; dilation; funnel configuration; membrane relationship; serial trajectory.
Safety and biological eligibility	Contractions or labor; membrane status; clinically significant infection or inflammation; bleeding; fetal and maternal status; contraindications; reasons for proceeding, deferring, transferring, modifying, or discontinuing.
Operative state	B–R–C descriptors when applicable; membrane-reduction method and result; change in reducibility or rim adequacy during preparation; tissue attenuation, friability, continuity, and apparent ability to retain a bite.
Mechanical objective	Intended proximal support, containment, limitation of further dilation, preservation of residual tissue, distal closure, or distributed reinforcement.
Intended configuration	Named technique; planned number of sutures; planned number of axial support levels; intended position and function of each suture; intended inter-level separation; placement sequence; material and caliber; and any pre-existing retained suture to be preserved, removed, or incorporated into the planned configuration.
Achieved reconstruction	Number of sutures placed and retained; number of axial support levels achieved; position and function of each suture; measured or described inter-level separation; tissue engagement; membrane position during tightening; completeness of closure; tissue response; status of any pre-existing suture; and deviations from the intended configuration.
Procedural course and harms	Membrane rupture; bleeding; cervical or vaginal injury; tissue tearing or cutting; infection; technical failure; operative time; removal considerations; other complications.
Postoperative assessment	Clinical indication for assessment; imaging route, timing, plane, and landmarks when used; residual closed length; funnel and membrane relationship; relative positions of multiple sutures; suspected migration or complications. Quantitative geometry or elastography should be labeled research-oriented and obtained under a prespecified protocol with standardized acquisition and reliability assessment.
Operator and center context	Operator experience; center volume or expertise; relevant resources and co-interventions that may affect feasibility, fidelity, complications, or outcomes.

Abbreviations: B–R–C, bulging extent–membrane reducibility–cervical rim adequacy.
